# The Association Between Thyroid Hormones and Renal Function in Euthyroid Chinese Individuals: A Population-Based Cross-Sectional Study

**DOI:** 10.7759/cureus.55682

**Published:** 2024-03-06

**Authors:** Zheng-Xin Liu, Jin-Lin Lv, Yu-Luan Xiang, Wenbin Deng, Hong Huang, Yin-Hua Sun, Li-Hua Li

**Affiliations:** 1 Department of Gerontology, The First Affiliated Hospital of Dali University, Dali, CHN

**Keywords:** albumin-to-creatinine ratio, glomerular filtration rate, chronic kidney disease, renal function, thyroid hormone

## Abstract

Objective

This population-based cross-sectional study aimed to investigate the association between thyroid hormones and renal function in euthyroid Chinese individuals, as the relationship between thyroid hormones and renal function in this population remains unclear.

Methods

A total of 661 participants were included in the study after excluding individuals with thyroid diseases, incomplete clinical measurements, or those taking medications affecting thyroid function. Participants were categorized into three groups based on serum thyroid hormone and antibody levels. The study adjusted for covariates and assessed the glomerular filtration rate (GFR) and urine albumin-to-creatinine ratio (ACR) in relation to thyroid hormone levels.

Results

After adjusting for covariates, the study found a significant increase in GFR in the middle and highest tertiles of free triiodothyronine (FT3) and the highest tertile of total triiodothyronine (TT3). Serum FT3 and TT3 levels were significantly associated with GFR. Additionally, the study observed a significantly lower GFR in the highest tertile of thyroid-stimulating hormone (TSH) compared to the lowest tertile. However, thyroid hormone and antibody levels were not associated with the ACR. Furthermore, the highest tertiles of TT3 and total thyroxine (TT4) were associated with a decreased risk of chronic kidney disease (CKD).

Conclusion

In our study among euthyroid Chinese individuals, we observed a significant association between thyroid function and GFR. Specifically, lower FT3, TT3, and higher TSH were associated with reduced GFR, indicating a potential role for thyroid hormones in maintaining renal function. Furthermore, lower levels of TT3 and TT4 were associated with an increased risk of CKD. These findings suggest a direct link between thyroid and renal function, even in euthyroid individuals, emphasizing the need for further investigation to elucidate the underlying mechanisms and potential therapeutic implications.

## Introduction

Chronic kidney disease (CKD) has become a global public health concern, and its prevalence increases with age [1]. In China, ≈10.8% of the population has CKD [1]. CKD is often accompanied by an increased risk of cardiovascular disease and mortality [2], which adversely affects physical and mental health and imposes a heavy social and economic burden. In developing countries, including China, primary glomerulonephritis is the most common cause of CKD [3]. Other common causes of CKD include diabetes and hypertension. However, interventions for these diseases cannot completely delay further renal function decline. Therefore, it is important to identify and address other factors that lead to CKD progression.

Numerous recent studies have reported that the kidney is an important target organ for thyroid hormone action [4-6]. Thyroid dysfunction is common in patients with CKD, and its prevalence increases with the severity of renal insufficiency. A German chronic kidney disease study showed an increased risk of adverse renal events and all-cause mortality in patients with CKD who have mild-to-moderately abnormal thyroid function [7]. Thyroid dysfunction, including subclinical hyper- and hypothyroidism, can cause renal impairment and an increased risk of CKD [5,8-11]. Furthermore, most renal manifestations of thyroid dysfunction are reversible following treatment [4,6].

Although some studies have examined the relationship between thyroid function and renal function, most studies were limited to patients with thyroid dysfunction [5,8,12] or type 2 diabetes [13]. Further, some studies only used simple indices [14] to evaluate thyroid and renal function. Other studies have suggested that thyroid hormone levels, even those within the normal reference range, can affect kidney function [14-17]. However, the relationship between thyroid function and renal function in the general Chinese population remains unclear [15]. Therefore, this study aimed to evaluate the relationships among thyroid hormone levels (including thyroid antibody levels), renal function (based on the estimated glomerular filtration rate (GFR) and urine albumin-to-creatinine ratio (ACR)), and CKD within euthyroid Chinese individuals.

## Materials and methods

Study participants

The data used in this cross-sectional study were obtained from an ongoing population study on cardiovascular risk factors in Dali, Yunnan Province, China [18]. From October to December 2018, we invited all residents aged ≥18 from two communities in Dali to participate in the study. After excluding participants with thyroid diseases, incomplete clinical measurements, and those taking medications that can affect thyroid function, including amiodarone, 661 participants were included in the present analysis. The study protocol was approved by the Institutional Review Board (IRB) of Dali University (Approval No: DU2018-3-8). This approval encompasses all ethical considerations pertaining to the study as per the university's guidelines and international research standards. All participants provided written informed consent.

Fieldwork

Two experienced physicians administered a standardized questionnaire to collect information regarding age, gender, medical history, smoking habits, alcohol intake, and medication. Furthermore, blood pressure (BP), pulse rate, body height, and weight were measured. Sitting BP in the nondominant arm was measured five consecutive times using a validated mercury sphygmomanometer, with one-minute intervals between measurements. The average of the five readings was included in the statistical analysis. Hypertension was defined by a systolic BP of ≥140 mmHg, a diastolic BP of ≥90 mmHg, or the use of antihypertensive drugs. Body mass index (BMI) was calculated by dividing the body weight in kilograms by the square of height in meters. Venous blood samples were obtained after overnight fasting to measure the serum levels of thyroid hormones, serum creatinine (Scr), plasma glucose, total cholesterol, and other biochemical markers. Diabetes mellitus was defined by a fasting plasma glucose level of ≥7.0 mmol/L, a hemoglobin A1c level of ≥6.5%, or the use of antidiabetic agents. Additionally, fasting morning spot urine samples were collected to determine the ACR. All blood and urine samples were tested in the Laboratory Department of the First Affiliated Hospital of Dali University using standard methods.

Thyroid function measurement

Serum thyroid hormone levels were measured, and autoantibodies were detected through electrochemiluminescence immunoassays. In our hospital, the normal ranges of thyroid-stimulating hormone (TSH), free thyroxine (FT4), free triiodothyronine (FT3), total T4 (TT4), and total T3 (TT3) levels are 0.35-4.94 μIU/mL, 9-26 pmol/L, 1.34-6.79 pmol/L, 45-133 nmol/L, and 0.66-2.3 nmol/L, respectively. Further, we measured the levels of thyroid antibodies such as thyroglobulin antibody (TGAb), thyroid microsomal antibody (TMAb), and thyroid peroxidase antibody (TPOAb).

Renal function measurement and definition of CKD

Renal function was assessed based on the ACR and GFR. The GFR was estimated using the CKD-Epidemiology Collaboration formula based on the participants' Scr level, age, and gender.

For women: 1) if Scr ≤0.7 mg/dL, 

$GFR= 144 * (\frac{Scr}{0.7})^{-0.329} * (0.993)^{age}$

2) if Scr＞0.7mg/dL, 

$GFR= 144 * (\frac{Scr}{0.7})^{-1.209}*(0.993)^{age}$

For men: 1) if Scr ≤0.9 mg/dL, 

$GFR = 141 * (\frac{Scr}{0.9})^{-0.411} * (0.993)^{age}$

2) if Scr＞0.9 mg/dL, 

$GFR = 141*(\frac{Scr}{0.9})^{-1.209}*(0.993)^{age}$ .

CKD was defined by a GFR of < 60 mL/min/1.73 m^2^ or the presence of proteinuria (ACR ≥ 30 mg/g) [19]. 

Statistical analysis

We utilized EmpowerStats software V5.0 for database management and statistical analysis. The Shapiro-Wilk test was employed to assess data normality. Data that adhered to a normal distribution is represented as mean ± standard deviation and was evaluated using an independent samplet-test. Conversely, data that did not adhere to a normal distribution is expressed as median (interquartile range) and was analyzed using a nonparametric test. Qualitative data is expressed as numbers (proportions) and was analyzed using the Chi-squared test.

Thyroid hormone levels were examined as either continuous or categorical variables, divided into tertiles as follows: serum FT3 (1.78-3.85, 3.86-4.69, 4.70-6.79 pmol/L), serum FT4 (9.01-15.04, 15.06-18.72, 18.73-25.98 pmol/L), serum TSH (0.14-0.80, 0.81-1.54, 1.56-27.75 IU/mL), serum TT3 (0.51-0.90, 0.91-1.20, 1.21-2.31 nmol/L), serum TT4 (45.06-75.35, 75.39-89.04, 89.21-132.97 nmol/L), serum TGAb (0.76-15.37, 15.41-23.88, 23.92-1086.10 IU/mL), serum TMAb (0.08-0.59, 0.60-0.98, 0.99-88.87 IU/mL), and serum TPOAb (0.62-5.64, 5.65-7.99, 8.00-190.91 S/CO).

Using the lowest tertile as a reference, we conducted univariate and multivariate-adjusted linear regression analyses to investigate the correlation of thyroid hormone levels with the GFR and ACR, represented using β and the 95% confidence interval (CI). Additionally, we executed univariate and multivariate-adjusted binary logistic regression to examine the correlation between thyroid function and the risk of CKD, which is represented using the odds ratio (OR) and 95% CI. The threshold for statistical significance was set at p<0.05.

## Results

Characteristics of the study population

Among the 661 participants, 421 (63.7%) were women, 209 (31.6%) had hypertension, and 72 (10.9%) had diabetes mellitus. Table 1 shows the characteristics of the study participants according to their gender. Current smoking status, alcohol intake, hypertension, and diabetes mellitus were more common in men than in women. Compared with women, men had a higher mean age, BMI, triglyceride levels, low-density lipoprotein cholesterol levels, Scr levels, and systolic and diastolic BPs (all p<0.05). Compared with women, men had lower GFRs and lower levels of high-density lipoprotein cholesterol, TT3, TSH, and TGAb (all p≤0.05). There were no significant between-gender differences in the levels of serum total cholesterol, FT3, FT4, TT4, TMAb, TPOAb, ACR, or pulse rate (p>0.05).

**Table 1:** Characteristics of the study population Values represent the mean ± standard deviation, median (interquartile range), or number of study participants, n, and percentage (%). LDL-C - low-density lipoprotein cholesterol; HDL-C - high-density lipoprotein cholesterol; FT3 - free T3; FT4- free T4; TT3, total triiodothyronine; TT4 - total thyroxine; TSH - thyroid-stimulating hormone; TGAb - thyroglobulin antibody; TMAb - thyroid microsomal antibody; TPOAb - thyroid peroxidase antibody; GFR - glomerular filtration rate; ACR - urine albumin-to-creatinine ratio

Variables	Men (n=240)	Women (n=421)	t/x^2^	p-value
Age, years	53.43 ± 13.05	51.24 ± 11.98	2.18	0.029
Body mass index, kg/m^2^	24.53 ± 3.38	23.62 ± 3.31	3.38	<0.001
Total cholesterol, mmol/L	4.82 ± 1.01	4.84 ± 0.91	-0.14	0.889
Triglyceride, mmol/L	1.70 (1.18–2.43)	1.43 (1.03–1.94)	3.04	<0.001
LDL-C, mmol/L	2.68 ± 0.74	2.53 ± 0.67	2.71	0.007
HDL-C, mmol/L	1.32 ± 0.22	1.44 ± 0.23	-6.28	<0.001
FT3, pmol/L	4.28 (3.57–4.96)	4.30 (3.61–4.97)	-0.85	0.397
FT4, pmol/L	16.69 (13.42–19.50)	16.80 (14.48–19.83)	-1.84	0.066
TT3, nmol/L	1.00 (0.80–1.28)	1.07 (0.85–1.31)	-2.17	0.031
TT4, nmol/L	80.72 (69.82–91.63)	82.47 (73.28–94.20)	-1.36	0.174
TSH, mIU/mL	0.97 (0.56–1.52)	1.28 (0.77–2.20)	-2.33	0.020
TGAb, IU/mL	17.13 (11.95–22.95)	19.73 (14.51–63.74)	-3.10	0.004
TMAb, IU/mL	0.78 (0.48–1.04)	0.80 (0.52–1.24)	-1.05	0.295
TPOAb, S/CO	6.16 (5.21–8.11)	6.90 (5.40–11.84)	-1.92	0.072
Serum creatinine, mmol/L	88.42 ± 17.84	63.33 ± 10.13	23.07	<0.001
GFR, mL/min/1.73 m^2^	87.13 ± 18.09	94.82 ± 18.38	-5.21	<0.001
ACR, mg/g	5.44 (3.52–14.36)	8.77 (4.04–15.57)	0.82	0.412
Systolic blood pressure, mmHg	122.48 ± 17.52	117.04 ± 17.75	3.80	<0.001
Diastolic blood pressure, mmHg	80.02 ± 11.92	75.84 ± 10.81	4.59	<0.001
Pulse rate, mmHg	71.28±9.31	72.39±8.52	-1.56	0.120
Current smoking, n (%)	136 (56.67%)	6 (1.43%)	276.55	<0.001
Alcohol intake, n (%)	71 (29.58%)	8 (1.90%)	111.32	<0.001
GFR<60 mL/min/1.73 m^2^, n (%)	14 (5.83%)	9 (2.14%)	6.22	0.013
Diabetes mellitus, n (%)	39 (16.25%)	33 (7.84%)	11.14	<0.001
Hypertension, n (%)	98 (40.83%)	111 (26.37%)	14.80	<0.001
Current antihypertensive treatment, n (%)	55 (22.92%)	74 (17.58%)	2.78	0.096

Association of thyroid function and antibody levels with the GFR and ACR

**Figure 1:**
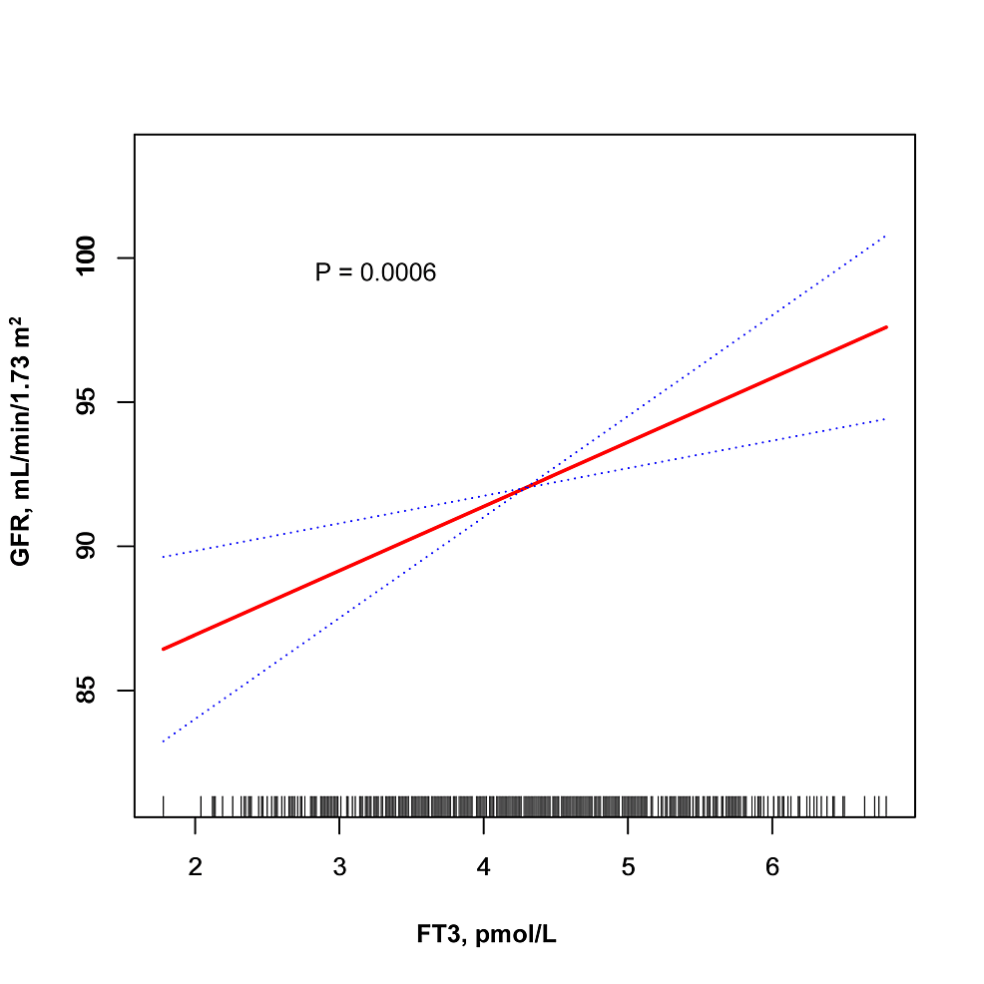
Smooth curve fitting between FT3 levels and the estimated glomerular filtration rate The values were adjusted for age, gender, body mass index, current smoking, alcohol intake, diabetes mellitus, hypertension, and antihypertensive treatment. The solid red line in the graph represents the trend line, and the blue dashed lines represent the 95% confidence interval. The short lines positioned along the x-axis denote distinct values of the FT3 levels. FT3 - free T3; GFR - glomerular filtration rate

**Figure 2:**
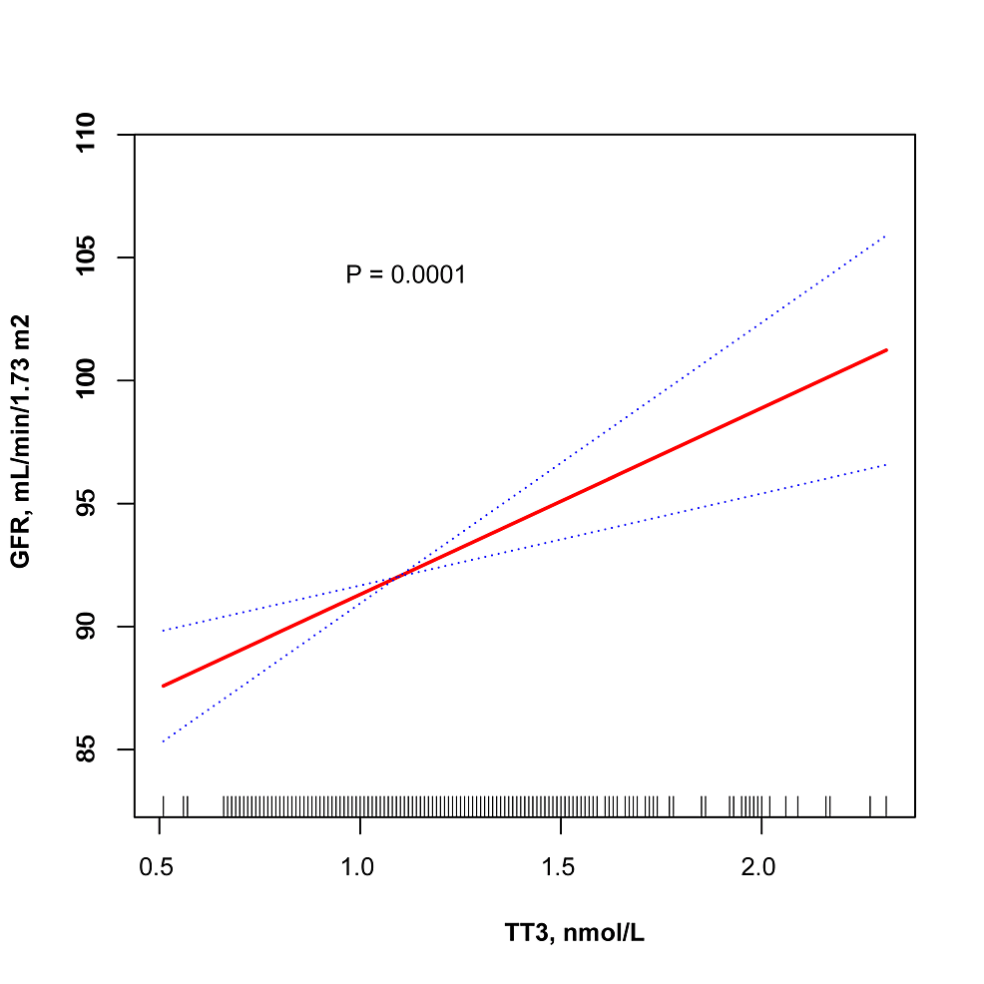
Smooth curve fitting between TT3 levels and the estimated glomerular filtration rate The values were adjusted for age, gender, body mass index, current smoking, alcohol intake, diabetes mellitus, hypertension, and antihypertensive treatment. The solid red line in the graph represents the trend line, and the blue dashed lines represent the 95% confidence interval. The short lines positioned along the x-axis denote distinct values of the TT3 levels. FT3 - free T3; GFR - glomerular filtration rate

Table 2 details the relationship between thyroid function, antibody levels, and GFR, adjusted for various covariates. We observed that GFR increased with higher tertiles of FT3 and TT3. Specifically, the highest tertiles of FT3 and TT3 showed a significant increase in GFR compared to the lowest tertiles. No significant differences were noted in GFR across the tertiles of TSH and other thyroid hormones and antibodies. The regression analyses confirmed a positive association between FT3, TT3, and GFR, as visualized in Figure 1 and Figure 2. Table 3 examines the association between thyroid function, antibody levels, and ACR, revealing no significant correlations. For precise β values, confidence intervals, and P-values, please refer to the respective tables and figures.

**Table 2:** Association of thyroid function and antibody levels with the glomerular filtration rate Age, gender, body mass index, current smoking, alcohol intake, diabetes mellitus, hypertension, and antihypertensive treatment were adjusted in the adjusted model. FT3 - free T3; FT4 - free T4; TT3 - total triiodothyronine; TT4 - total thyroxine; TSH - thyroid-stimulating hormone; TGAb - thyroglobulin antibody; TMAb - thyroid microsomal antibody; TPOAb - thyroid peroxidase antibody

	Crude model		Adjusted model	
	β (95% CI)	p-value	β (95% CI)	p-value
FT3, pmol/L	2.46 (1.00, 3.91)	0.0010	2.23 (0.96, 3.50)	0.0006
FT3 Tertile				
Low	0		0	
Middle	3.75 (0.29, 7.21)	0.0342	3.21 (0.19, 6.24)	0.0377
High	5.70 (2.25, 9.15)	0.0013	5.49 (2.48, 8.50)	0.0004
FT4, pmol/L	0.23 (−0.12, 0.58)	0.1987	0.14 (−0.17, 0.45)	0.3654
FT4 Tertile				
Low	0		0	
Middle	1.62 (−1.87, 5.10)	0.3627	1.68 (−1.39, 4.75)	0.2844
High	0.81 (−2.67, 4.29)	0.6483	0.36 (−2.70, 3.42)	0.8172
TT3, nmol/L	6.26 (1.87, 10.65)	0.0053	7.58 (3.74, 11.42)	0.0001
TT3 Tertile				
Low	0		0	
Middle	0.30 (−3.17, 3.77)	0.8662	1.21 (−1.84, 4.26)	0.4378
High	4.41 (0.95, 7.87)	0.0127	5.15 (2.13, 8.17)	0.0009
TT4, nmol/L	0.03 (−0.06, 0.11)	0.5779	0.07 (−0.01, 0.15)	0.0895
TT4 Tertile				
Low	0		0	
Middle	0.86 (−2.63, 4.35)	0.6288	1.96 (−1.11, 5.03)	0.2105
High	1.20 (−2.28, 4.69)	0.4983	2.21 (−0.89, 5.32)	0.1627
TSH, mIU/mL	−0.52 (−1.25, 0.21)	0.1637	−0.43 (−1.07, 0.22)	0.1935
TSH Tertile				
Low	0		0	
Middle	−0.61 (−4.09, 2.86)	0.7299	−1.84 (−4.89, 1.22)	0.2388
High	−3.80 (−7.28, -0.32)	0.0326	−4.09 (−7.21, −0.98)	0.0103
TGAb, IU/mL	−0.00 (−0.01, 0.01)	0.4628	−0.01 (−0.01, 0.00)	0.1506
TGAb Tertile				
Low	0		0	
Middle	1.12 (−2.36, 4.61)	0.5276	1.82 (−1.22, 4.87)	0.2409
High	1.64 (−1.84, 5.12)	0.3573	1.42 (−1.67, 4.51)	0.3669
TMAb, IU/mL	−0.11 (−0.36, 0.14)	0.3911	−0.06 (−0.28, 0.15)	0.5703
TMAb Tertile				
Low	0		0	
Middle	2.95 (−0.51, 6.42)	0.0955	1.49 (−1.57, 4.55)	0.3411
High	−1.58 (−5.04, 1.89)	0.3720	−1.48 (−4.52, 1.57)	0.3427
TPOAb, S/CO	0.01 (−0.03, 0.04)	0.7677	0.00 (−0.03, 0.03)	0.9373
TPOAb Tertile				
Low	0		0	
Middle	−0.60 (−4.09, 2.88)	0.7348	−0.80 (−3.86, 2.27)	0.6109
High	0.72 (−2.77, 4.22)	0.6855	0.01 (−3.07, 3.09)	0.9966

**Table 3:** Association of thyroid function and antibody levels with the urine albumin-to-creatinine ratio Age, gender, body mass index, current smoking, alcohol intake, diabetes mellitus, hypertension, and antihypertensive treatment were adjusted in the adjusted model. FT3 - free T3; FT4 - free T4; TT3 - total triiodothyronine; TT4 - total thyroxine; TSH - thyroid-stimulating hormone; TGAb - thyroglobulin antibody; TMAb - thyroid microsomal antibody; TPOAb - thyroid peroxidase antibody

	Crude model		Adjusted model	
	β (95% CI)	p-value	β (95% CI)	p-value
FT3, pmol/L	1.86 (−4.61, 8.34)	0.573	2.89 (−3.40, 9.18)	0.3677
FT3 Tertile				
Low	0		0	
Middle	4.31 (−11.11, 19.73)	0.5839	6.18 (−8.79, 21.16)	0.4186
High	9.17 (−6.18, 24.52)	0.2421	10.44 (−4.45, 25.34)	0.1698
FT4, pmol/L	1.62 (0.07, 3.17)	0.0406	1.21 (−0.30, 2.73)	0.1168
FT4 Tertile				
Low	0		0	
Middle	6.03 (−9.33, 21.40)	0.4418	−0.92 (−15.96, 14.12)	0.9047
High	16.82 (1.49, 32.15)	0.0319	11.87 (−3.11, 26.84)	0.1209
TT3, nmol/L	0.82 (−18.68, 20.32)	0.9344	4.69 (−14.34, 23.72)	0.6293
TT3 Tertile				
Low	0		0	
Middle	10.15 (−5.27, 25.57)	0.1974	6.78 (−8.32, 21.89)	0.3791
High	3.99 (−11.36, 19.34)	0.6106	5.27 (−9.68, 20.23)	0.4897
TT4, nmol/L	0.13 (−0.26, 0.52)	0.4995	−0.07 (−0.45, 0.32)	0.736
TT4 Tertile				
Low	0		0	
Middle	3.87 (−11.52, 19.27)	0.6221	0.17 (−14.89, 15.24)	0.9819
High	6.75 (−8.64, 22.15)	0.3903	−1.81 (−17.08, 13.47)	0.8168
TSH, mIU/mL	0.69 (−2.53, 3.91)	0.6742	1.08 (−2.07, 4.22)	0.5026
TSH Tertile				
Low	0		0	
Middle	1.86 (−13.55, 17.28)	0.8127	3.59 (−11.44, 18.63)	0.6396
High	7.03 (−8.40, 22.46)	0.3724	10.18 (−5.14, 25.51)	0.1933
TGAb, IU/mL	0.00 (−0.03, 0.04)	0.8137	0.01 (−0.03, 0.04)	0.6253
TGAb Tertile				
Low	0		0	
Middle	12.63 (−2.73, 27.98)	0.1075	12.50 (−2.40, 27.40)	0.1005
High	8.26 (−7.11, 23.64)	0.2925	7.88 (−7.27, 23.03)	0.3086
TMAb, IU/mL	0.02 (−1.09, 1.12)	0.9782	0.12 (−0.95, 1.19)	0.8296
TMAb Tertile				
Low	0		0	
Middle	−9.97 (−25.33, 5.40)	0.204	−3.71 (−18.75, 11.33)	0.6289
High	−1.80 (−17.18, 13.58)	0.8185	1.37 (−13.62, 16.35)	0.8583
TPOAb, S/CO	0.03 (−0.13, 0.20)	0.6976	0.05 (−0.11, 0.22)	0.5253
TPOAb Tertile				
Low	0		0	
Middle	−6.21 (−21.60, 9.17)	0.4288	−8.07 (−23.07, 6.94)	0.2925
High	−1.92 (−17.38, 13.55)	0.8083	−1.18 (−16.28, 13.92)	0.8779

Association of thyroid function and antibody levels with CKD

In an initial analysis without adjusting for covariates, higher levels of TT3 were linked to a significantly reduced risk of CKD. This association remained strong even after adjusting for covariates such as age, gender, BMI, current smoking, alcohol intake, diabetes, hypertension, and antihypertensive treatment. Similarly, the highest levels of TT4 were also related to a lower risk of CKD. However, no significant associations were found between other thyroid hormones or antibody levels and CKD. Detailed statistical outcomes, including odds ratios and confidence intervals, are presented in Table 4.

**Table 4:** Association of thyroid function and antibody levels with the risk of chronic kidney disease Age, gender, body mass index, current smoking, alcohol intake, diabetes mellitus, hypertension, and antihypertensive treatment were adjusted in the adjusted model. FT3 - free T3; FT4 - free T4; TT3 - total triiodothyronine; TT4 - total thyroxine; TSH - thyroid-stimulating hormone; TGAb - thyroglobulin antibody; TMAb - thyroid microsomal antibody; TPOAb - thyroid peroxidase antibody

Exposure	Crude model		Adjusted model	
	OR (95% CI)	p-value	OR (95% CI)	p-value
FT3 group				
Low	1.0		1.0	
Middle	0.63 (0.24, 1.65)	0.3445	1.03 (0.34, 3.15)	0.9526
High	0.44 (0.15, 1.28)	0.1317	0.40 (0.11, 1.44)	0.1619
FT3 group trend	0.65 (0.37, 1.12)	0.1205	0.66 (0.35, 1.22)	0.1829
FT4 group				
Low	1.0		1.0	
Middle	1.15 (0.41, 3.22)	0.7929	0.97 (0.30, 3.17)	0.9559
High	1.14 (0.41, 3.21)	0.7998	1.21 (0.36, 4.01)	0.7585
FT4 group trend	1.02 (0.89, 1.15)	0.8050	1.03 (0.88, 1.19)	0.7486
TSH group				
Low	1.0		1.0	
Middle	1.37 (0.43, 4.40)	0.5923	1.99 (0.53, 7.47)	0.3088
High	2.22 (0.76, 6.50)	0.1454	2.42 (0.72, 8.07)	0.1516
TSH group trend	1.45 (0.90, 2.32)	0.1230	1.42 (0.85, 2.39)	0.1835
TT3 group				
Low	1.0		1.0	
Middle	1.22 (0.51, 2.88)	0.6543	1.46 (0.51, 4.24)	0.4824
High	0.10 (0.01, 0.75)	0.0254	0.05 (0.01, 0.48)	0.0091
TT3 group trend	0.10 (0.01, 0.65)	0.0160	0.05 (0.00, 0.42)	0.0062
TT4 group				
Low	1.0		1.0	
Middle	0.69 (0.26, 1.85)	0.4603	0.45 (0.14, 1.51)	0.1976
High	0.59 (0.21, 1.64)	0.3092	0.24 (0.07, 0.85)	0.0271
TT4 group trend	0.98 (0.95, 1.02)	0.3074	0.95 (0.92, 1.00)	0.0293
TGAb group				
Low	1.0		1.0	
Middle	1.00 (0.39, 2.57)	1.0000	0.56 (0.18, 1.73)	0.3123
High	0.54 (0.18, 1.65)	0.2803	0.32 (0.09, 1.15)	0.0812
TGAb group trend	0.99 (0.98, 1.00)	0.2339	0.99 (0.98, 1.00)	0.1366
TMAb group				
Low	1.0		1.0	
Middle	0.39 (0.12, 1.26)	0.1151	0.66 (0.18, 2.47)	0.5392
High	0.89 (0.36, 2.24)	0.8068	1.03 (0.36, 2.98)	0.9508
TMAb group trend	1.02 (0.46, 2.25)	0.9627	1.07 (0.46, 2.52)	0.8737
TPOAb group				
Low	1.0		1.0	
Middle	1.81 (0.66, 4.98)	0.2520	2.90 (0.83, 10.16)	0.0956
High	0.98 (0.31, 3.08)	0.9679	1.30 (0.35, 4.83)	0.6972
TPOAb group trend	0.98 (0.90, 1.06)	0.5474	0.98 (0.90, 1.07)	0.6839

## Discussion

This population-based study on euthyroid Chinese participants revealed a significant positive correlation of serum FT3 and TT3 levels with the GFR. Moreover, serum TSH levels were negatively correlated with the GFR. Serum TT3 and TT4 levels were negatively associated with the risk of CKD. These findings suggest that thyroid function in euthyroid participants is significantly associated with GFR and the risk of CKD.

Several studies have described the mechanisms through which thyroid hormones act on the kidneys [6,20]. Thyroid hormones directly or indirectly affect renal function by affecting renal growth and development, hemodynamics, GFR, and the water and electrolyte balance [21]. In hyperthyroidism, thyroid hormones can increase the heart rate, cardiac output, and nitric oxide synthesis, decrease peripheral vascular resistance, and dilate the renal vasculature [6]. In addition, thyroid hormone can increase the plasma levels of renin, angiotensin II, and angiotensin-converting enzymes and enhance the activity of the renin-angiotensin-aldosterone system (RAAS) [10]. Therefore, glomerular afferent arterioles dilate, efferent arterioles contract, and intraglomerular pressure increases, which all lead to an increase in renal blood flow and GFR. Additionally, hyperfiltration in patients with hyperthyroidism may lead to increased 24-hour proteinuria [22]; further, renal tubular damage may be caused by ultrafiltration, hypertrophy, and hyperplasia [22].

Conversely, in hypothyroidism, there is decreased myocardial contractility, cardiac output, and renal blood flow; decreased synthesis of vascular endothelial relaxation factor, resulting in arterial stiffness; increased systemic vascular resistance; and reduced sensitivity to β-adrenergic receptors and decreased renin expression and release, resulting in decreased RAAS activity [22]. The aforementioned mechanisms reduce the GFR and increase Scr [6]. Additionally, increased permeability of glomerular capillaries to proteins in hypothyroidism results in proteinuria [22]. However, even lower thyroid function within the normal range is associated with reduced renal blood flow [23]. We previously found that serum FT4 levels were associated with high peripheral and central BP and an increased risk of arterial stiffness in men than in women; moreover, serum FT3 levels were positively associated with a high pulse rate [18]. The influence of thyroid hormones on renal function may be indirectly caused by the influence of thyroid hormones on BP and heart rate.

A previous cross-sectional Chinese study found that TSH levels were negatively correlated with the GFR, which suggested that an increased TSH level may be an independent risk factor for CKD in a middle-aged Chinese population with normal blood glucose and thyroid function [16]. Moreover, Wang et al. analyzed data from 10,859 euthyroid Chinese individuals who underwent annual regular health checkups and found that TSH levels were negatively associated with the GFR [14]. Even within the normal range, higher TSH levels have been associated with a decreased GFR and an increased prevalence of CKD [17,24-25]. A retrospective study of Chinese adults observed a negative correlation between FT3 levels and the risk of stages 1-4 CKD. Moreover, an increase in FT4 and a decrease in TSH was associated with a decreased risk of stage 5 CKD, which suggests that serum FT3 can be used as an early biomarker for CKD, while FT4 and TSH can be used as late biomarkers for CKD [15]. A previous study on patients with type 2 diabetes found that higher TSH and FT4 (or TT4), lower FT3 (or TT3), and TGAb positivity were associated with a higher ACR and lower GFR.

Additionally, increased TSH and FT4 (or TT4) and decreased FT3 (or TT3) levels were associated with the prevalence of kidney disease [26]. Even within the normal reference range, decreased FT3 levels were associated with a decreased GFR [26]. The relationship between FT3 levels and the GFR is independent of the applied GFR equation [27]. Das et al. reported that serum TSH levels are positively correlated with microalbuminuria in patients with diabetes, even within the euthyroid range [28]. Another prospective study showed that higher levels of FT4, but not TSH and FT3, were associated with an increased risk of CKD, a rapid decline in the GFR, and an increased risk of complications [29]. Consistent with the aforementioned studies, our findings demonstrated relationships among the levels of TSH and FT3, GFR, and the risk of CKD. However, there have been inconsistent reports regarding the relationship between FT4 levels and renal function [14,30]. Wang et al. found that FT4 levels were not associated with the GFR but were positively associated with the risk of CKD [14]. A cross-sectional analysis of data from 7933 individuals aged 20-93 years in the Berlin Aging Study II and Study of Health in Pomerania found that both high and low serum FT4 levels were associated with a decreased GFR [30]. In the present study, there was no correlation between thyroid hormone and antibody levels with ACR or between thyroid antibody levels and GFR. In addition to the effects of TSH and FT3 on renal function, our findings suggest a positive correlation between serum TT3 levels and the GFR, as well as a negative correlation of serum TT3 and TT4 levels with the risk of CKD.

There have been inconsistent reports regarding the relationship between thyroid hormones and renal function in euthyroid individuals. For example, Meuwese et al. reported that in the general older population, high TSH levels and low FT4 and FT3 levels were associated with decreased renal function but no further decrease in renal function over time [12]. Similarly, other studies have suggested that patients with hypothyroidism or subclinical hypothyroidism have a lower GFR than euthyroid patients, while those with hyperthyroidism or subclinical hyperthyroidism have a higher GFR than euthyroid patients [31]. However, decreased thyroid function is not associated with worsening renal function [31]. The relationship observed in this cross-sectional study could be attributed to renal insufficiency leading to abnormal thyroid function [31]. Decreased thyroid function is closely related to decreased renal function, which may be due to CKD-induced hypofunction of the hypothalamic-pituitary-thyroid axis, which is termed as "non-thyroid disease" (abnormal thyroid hormone levels in the absence of hypothalamic-pituitary-thyroid disease) [32]. Contrastingly, primary hypothyroidism may lead to decreased renal function. Therefore, further research on the relationship between thyroid and renal function is necessary.

This study has some limitations. First, we could not establish a causal relationship between thyroid hormone levels and the risk of CKD since this was a cross-sectional study. Further prospective studies are warranted to determine whether a causal relationship exists. Second, the relatively small sample size may have biased our findings. Third, thyroid function and the ACR, which are influenced by multiple factors and may fluctuate over time, were measured only once in our study. Finally, our study included a few patients with hypertension who were receiving antihypertensive therapy and certain medications, such as β-blockers, which may affect thyroid hormone levels and function. Furthermore, the use of angiotensin-converting enzyme inhibitors and angiotensin receptor blockers may impact the ACR. Although we adjusted for current antihypertensive treatment in our multivariate analyses, we cannot entirely rule out the potential effect of antihypertensive therapy on thyroid hormone or ACR levels.

Despite the aforementioned limitations, we found that even within the normal range, thyroid function was related to the GFR, which has clinical significance in terms of the prediction and treatment of CKD. In clinical practice, thyroid function is easy to measure; accordingly, thyroid hormone levels may be useful in predicting the occurrence and progression of CKD. Additionally, supplementing thyroid hormones and improving thyroid function may help slow down renal function deterioration.

## Conclusions

Our study demonstrated a significant association between thyroid function and GFR as well as the risk of CKD in euthyroid Chinese individuals. Specifically, we observed a positive correlation between thyroid function and GFR, indicating a potential protective role of thyroid hormones in maintaining renal function. Conversely, we found a negative correlation between thyroid function and CKD risk, suggesting that thyroid dysfunction may increase the vulnerability to CKD development.

However, it is important to note that our findings do not establish a causal relationship between thyroid and renal function. Future prospective studies are necessary to confirm the directionality of this association. Additionally, it would be beneficial to explore whether thyroid hormone supplementation can confer benefits to patients with CKD and decreased thyroid function.

Overall, our findings highlight the significance of thyroid function in renal health and underscore the need for further research to elucidate the underlying mechanisms and potential therapeutic implications.
